# Selective citation in the literature on swimming in chlorinated water and childhood asthma: a network analysis

**DOI:** 10.1186/s41073-017-0041-z

**Published:** 2017-10-02

**Authors:** Bram Duyx, Miriam J. E. Urlings, Gerard M. H. Swaen, Lex M. Bouter, Maurice P. Zeegers

**Affiliations:** 1 Care and Public Health Research Institute (School CAPHRI), Maastricht University, Maastricht, The Netherlands; 2 Nutrition and Translational Research in Metabolism (School NUTRIM), Maastricht University, Maastricht, The Netherlands; 3 Department of Epidemiology and Biostatistics, VU University Medical Center, Amsterdam, The Netherlands; 4 Department of Philosophy, Faculty of Humanities, Vrije Universiteit, Amsterdam, The Netherlands

**Keywords:** Citation analysis, Knowledge generation, Selective citation, Pool chlorine hypothesis, Childhood asthma, Swimming pool, Chlorinated pool

## Abstract

**Background:**

Knowledge development depends on an unbiased representation of the available evidence. Selective citation may distort this representation. Recently, some controversy emerged regarding the possible impact of swimming on childhood asthma, raising the question about the role of selective citation in this field. Our objective was to assess the occurrence and determinants of selective citation in scientific publications on the relationship between swimming in chlorinated pools and childhood asthma.

**Methods:**

We identified scientific journal articles on this relationship via a systematic literature search. The following factors were taken into account: study outcome (authors’ conclusion, data-based conclusion), other content-related article characteristics (article type, sample size, research quality, specificity), content-unrelated article characteristics (language, publication title, funding source, number of authors, number of affiliations, number of references, journal impact factor), author characteristics (gender, country, affiliation), and citation characteristics (time to citation, authority, self-citation). To assess the impact of these factors on citation, we performed a series of univariate and adjusted random-effects logistic regressions, with potential citation path as unit of analysis.

**Results:**

Thirty-six articles were identified in this network, consisting of 570 potential citation paths of which 191 (34%) were realized. There was strong evidence that articles with at least one author in common, cited each other more often than articles that had no common authors (odds ratio (OR) 5.2, 95% confidence interval (CI) 3.1–8.8). Similarly, the chance of being cited was higher for articles that were empirical rather than narrative (OR 4.2, CI 2.6–6.7), that reported a large sample size (OR 5.8, CI 2.9–11.6), and that were written by authors with a high authority within the network (OR 4.1, CI 2.1–8.0). Further, there was some evidence for citation bias: articles that confirmed the relation between swimming and asthma were cited more often (OR 1.8, CI 1.1–2.9), but this finding was not robust.

**Conclusions:**

There is clear evidence of selective citation in this research field, but the evidence for citation bias is not very strong.

**Electronic supplementary material:**

The online version of this article (doi:10.1186/s41073-017-0041-z) contains supplementary material, which is available to authorized users.

## Background

The number of citations that a publication receives is often regarded as an indicator of scientific quality [1]. The rationale behind this is that high-quality work will lead to more citations by peer scientists compared to low-quality work. It can be questioned, however, whether it is scientific merit alone that drives the number of citations or whether other factors have an influence as well.

Due to the large and growing number of scientific articles in the biomedical domain and limitation of the maximum number of references in many journals, it is not always feasible to cite all available literature. Ideally, a representative sample should be cited, but it is unclear on which ground researchers select the articles they cite. If this selection is not representative, but instead associated with specific characteristics of the cited literature, we speak of selective citation.

Citation bias is a special case of selective citation [2]. It concerns the selective citation of studies based on their outcome. Citation bias results in disproportionate attention for a specific segment of the literature that is in line with a hypothesis, while publications with an alternative view are systematically ignored. This can lead to a false consensus that is not evidence-based [3]. For instance, it has been shown that biased citation of previous evidence shapes the conclusions of reviews [4]. Citation bias can also lead to research waste by influencing funding decisions and guiding research in a wrong direction [4, 5].

Citation analyses can be performed in several manners. Greenberg [3] performed a network analysis based on a specific scientific claim: the relationship between a muscle disease called inclusion body myositis and beta-amyloid proteins. After identifying all the literature on this claim, he compared the number of citations to supportive empirical publications with the number of citations to critical ones. Greenberg concluded that 94% of the citations were assigned to the supportive data [3]. Next to a claim-specific network, a network analysis can also be based on a specific journal or time period. For instance, Fanelli [5] performed a citation analysis across several research domains. Articles were included if they tested any hypothesis and were published between 2000 and 2007. It was found that positive studies received on average 32% more citations than negative studies. However, this percentage differed substantially between the scientific domains [5].

In the current study, we performed a claim-specific citation analysis. We focused on the relation between swimming in chlorinated water and the development of childhood asthma.

### Chlorinated water and asthma

The prevalence of childhood asthma is high in the developed world [6] and rising in the developing world [7]. Asthma patients are often recommended to swim because it has been shown that exercise can help to alleviate asthmatic symptoms (e.g., [8]). This recommendation has been endorsed by some health authorities and official guidelines worldwide (e.g., [9]).

In 2003, however, Bernard et al. postulated that swimming in chlorinated water could also increase the risk of developing asthma [10], especially in children. This can be explained as follows. Human beings secrete sweat and urine in swimming water, and both of these contain urea. A chemical reaction of urea with the free chlorine in chlorinated water leads to the generation of chloramines. In fact, it is these chloramines that are responsible for the typical smell of chlorinated swimming pools, not the chlorine itself. One of these chloramines, called trichloramine or nitrogen trichloride (NCl_3_), does not dissolve in water and will be released into the air [11, 12]. This substance can cause irritation to the eyes and lungs. The actual exposure to trichloramine depends on many factors like the level of free chlorine in the swimming water, whether the swimming pool is indoors or outdoors, ventilation, the number of people in the swimming pool, whether they have showered before, and the duration and intensity of the exercise. Children might be particularly prone to the adverse effects of trichloramine, as their lungs are still developing and relatively sensitive.

The pool chlorine hypothesis builds on this potential relationship between swimming in chlorinated water and the development of asthma in children. It postulates that the alleged rise in childhood asthma in developed countries can at least partly be explained by the popularity of indoor chlorinated pools [6] (but see also [7] for counter-evidence on the rise of asthma in the developed world). If swimming in chlorinated water indeed has an adverse effect on respiratory health, it would call into question the official recommendation for asthmatic patients to swim. It would also suggest that other methods should be applied for the disinfection of swimming pool water.

However, no consensus has yet been reached on the question whether swimming in chlorinated water does increase the likelihood of developing asthma, and as such, it is still an object of ongoing research. After all, it is hard to find conclusive evidence that would settle the debate in the form of a single study, even more so because most of the studies on humans are observational rather than experimental. In addition, asthma is difficult to diagnose and has been assessed in a variety of ways. It is therefore necessary to combine different sources of information to reach a representative overview of the literature and an evidence-based consensus.

In order to investigate how these different sources of information are combined in this field, we employed a citation analysis on all published literature investigating the claim that swimming in chlorinated water is related to the development of childhood asthma. In addition to Greenberg’s approach [3], other determinants than study outcome were also taken into account. Specifically, our aim was to investigate which determinants influenced the likelihood of being cited in the scientific literature on the relation between swimming in chlorinated water and the development of childhood asthma.

## Method

Prior to performing this citation network analysis, we described the methodology in a study protocol and stored it at an online repository [13]. Deviations from this protocol are mentioned in the digital Additional file 1. In brief, we applied a search strategy to the Web of Science Core Collection, identified relevant literature, downloaded these records with their reference lists, extracted data for each article, built a dataset with potential citations, and used specialized software to determine which citations had occurred. These steps will be explained in more detail below.

### Search strategy

The search strategy combined terms related to (a) the determinant (terms like chlorinated pool, swimming, trichloramine), (b) the health outcome (asthma diagnosis and symptoms, lung measures), and (c) the population age (children and adolescents). The exact search strategy can be found in Additional file 2.

Publications were identified via the Web of Science Core Collection. Identification of articles via reference list checking was not applied, as this could result in an overrepresentation of cited articles. The search was performed by BD and updated until 20 June 2017. There were no restrictions with regard to publication language or year.

Both empirical and non-empirical articles were included. Articles were included if they presented data or contained a statement on the association between swimming in indoor chlorinated pools and childhood asthma or other asthma-related health outcome measures. Articles on swimming as recommendation to asthmatic patients were excluded, as well as those on swimming pool accidents. We made some minor deviations with regard to the criteria as described in the study protocol, the updated list can be found in the Additional file 1. Selection of articles was conducted by two authors (BD and MJEU) followed by a consensus meeting. Agreement was reached in all cases.

### Data extraction

A range of variables were extracted from each included publication. Data extraction was performed by two authors (BD and MJEU), followed by a consensus meeting. Agreement was reached in all cases. In addition, we developed measures for the within-network authority of the authors and for the occurrence of self-citations. These extracted and developed variables were classified in three distinct categories: article characteristics (study outcome, other content-related, not content-related), author characteristics, and citation characteristics.

#### Article characteristics––study outcome

We differentiated between two ways of looking at study outcome: data-based conclusion and authors’ conclusion. Selective citation based on either of these classifications of study outcome would signify citation bias.

The *data-based conclusion* was based on the asthma diagnosis results as reported in the result sections of empirical articles. Asthma diagnosis was assessed in different ways in the various articles. We ranked these assessments in order of decreasing validity: (1) physician’s assessment, (2) self-assessment, (3) occurrence of asthma symptoms, (4) positive lung tests, and (5) blood tests indicative of increased lung permeability. A data-based conclusion was scored as positive if a statistically significant, positive relationship of swimming in chlorinated water with asthma diagnosis was reported. In case of contradicting results, we used the asthma diagnosis with the highest validity. For instance, if blood tests showed a positive relationship with swimming but the physician’s assessment did not, the data-based conclusion was scored as negative.

The *authors’ conclusion* was scored in a similar way, but then based on the authors’ interpretation of the results instead of the statistical significance of asthma diagnosis. The authors’ conclusion was extracted from the discussion or abstract of a publication.

#### Article characteristics––other content-related

The following variables were in this subcategory: article type (and study design), sample size, study quality, and specificity.

*Article type* was classified into empirical articles and non-empirical articles (narrative reviews and commentaries). For some analyses, empirical articles were further classified into *study design*: experimental studies and observational studies (such as cross-sectional, cohort, ecological, case control studies, case studies, and systematic reviews of observational studies).

*Sample size* concerned the number of underage participants in the articles (younger than 18). Narrative reviews had no sample size, the other study designs were classified in three fairly equal categories.

The *study quality* of cross-sectional designs was rated with the NIH National Heart Lung and Blood Institute’s assessment for cross-sectional designs [14]. According to this scale, articles could be classified as good, fair, or poor. Other designs were not rated since the vast majority of the empirical studies was cross-sectional and the other designs had a very low number of publications.

The *specificity* of the articles could vary. Some articles may deal only with the statement under investigation (i.e., the relationship between swimming in chlorinated water and the development of asthma in children), others were broader (e.g., the health effects of swimming in the general population). The higher the specificity of an article, the better this article would fit in the network. Specificity ranged from 1 (very broad) to 5 (highly specific). Specificity was assessed based on the title of the article.

#### Article characteristics––not content-related

The following variables were in this category: language (English or not), conclusiveness of the title, funding source, number of authors, number of affiliations, number of references, and journal impact factor. Title conclusiveness was coded as yes if a clear outcome was stated in the title (e.g., “swimming and asthma are related” or “(...) not related”), otherwise as no (e.g., “swimming and asthma”). Funding source was coded as non-profit (e.g., government or university), for profit, both, or not reported. Journal impact factor, in the publication year of the potentially cited article, was retrieved from the Journal Citation Reports (JCR) database.

#### Author characteristics

The following variables were in this category: gender of the corresponding author (assessed by first name and/or salutation), country of the corresponding author, and affiliation of the corresponding author. Affiliation was classified as government, university, industry, or other.

#### Citation characteristics

There were some variables that depend on the cited article as well as the citing article: time to citation, authority, and self-citation. (For clarification: when we write about cited articles, citing articles, and citation paths, we refer to potential citations that may or may not occur.)

*Time to citation* was the number of years between the publication date of the cited article and the submission date of the citing article. This variable was also used to determine the dataset of potential citation paths (see “Section 2.3” below).

As for the publication date, we used either the electronic publication date or the paper publication date, depending on which one was earlier. The average duration from submission to publication was 7 months in this network. Submission date was not always given. If submission date was missing, it was estimated by subtracting 7 months from an article’s publication date.

*Within-network authority* was a measure for the authority of the authors of a cited article within the network. It was calculated for each author and each year separately, by counting the number of within-network citations to all publications in which the author had been involved. As the number of citations is likely to increase each year, so does the author’s authority. Because we were interested in the authority at the moment of citation, the authority value of a cited article also depends on the publication year of the citing article. In case of multiple authors, we used the authority value of the author with the highest authority in that year.

A *self-citation* was defined as a citation between two articles that have at least one author in common.

### Statistical analysis

The dataset consisted of all potential citation paths between citing and cited articles. A potential citation path means that the cited article is published before submission of the citing article (i.e., *time to citation* has a positive value). The underlying assumption is that articles can only cite up to their submission date and can only be cited from their publication date onwards. This assumption was met for the entire network with one exception: one article had cited another article that was not yet published at the moment of submission of the citing article. The same authors were involved in both articles, which explains why they could be aware of the cited article before it was published. (This citation was not considered a potential citation and therefore excluded from our analyses.)

Our dependent variable was citation or, in other words, whether a potential citation path was used or not. We used the built-in algorithm of CitNetExplorer to determine whether a citation had occurred [15]. This algorithm makes use of reference lists that can be downloaded from the Web of Science Core Collection. The reference lists of all articles in the network were linked by the algorithm with the actual articles in the network. If possible, this linkage was done by DOI, a unique Digital Object Identifier assigned to most present-day articles; otherwise, it was based on a combination of first author’s surname, first author’s first initial, publication year, volume number, and first page number. Manual checking of the reference lists of the included articles showed that all classified citations were correct and that no citations were missed by the algorithm. The determinants of citation were the characteristics of the cited article as described above.

Since each article could cite multiple other articles, the potential citation paths were related. Therefore, we used a multilevel approach in which the potential citations were nested under the citing article. Specifically, we performed a univariate random-effects logistic regression for each determinant of citation. We repeated these analyses while adjusting for article type.

Where applicable, we also calculated whether the cited and the citing articles had the same characteristics (*concordance*). This would for instance be the case if positive articles would prefer to cite other positive articles and if negative articles would prefer to cite other negative articles. If citation would be based on the concordance of study outcome, it would be another sign of citation bias. To test if concordance on several characteristics has an impact on the likelihood of citation, univariate and adjusted fixed-effects logistic regression analyses were applied.

### Software

We used the built-in algorithm of CitNetExplorer 1.0.0 to extract the actual citations between articles. We used R 3.2.4 to create a dataset with all potential citation paths, based on the data extraction sheet and the actual citations, and also to calculate the within-network authority, self-citation score, and time to citation for each potential citation path. Finally, we used Stata 13.1 to analyze the results and VOSviewer 1.6.0 to assess the co-authorship networks. CitNetExplorer and VOSViewer were also used to visualize the network.

## Results

Thirty six articles on the relationship between swimming in chlorinated water and the development of childhood asthma, published between 2002 and 2015, were identified via the Web of Science [6, 10, 12, 16–48] (see Additional file 3). There were 191 actual citations between these articles, out of a total of 570 potential citation paths (34%). The characteristics of the publications are depicted in Table 1. Of the 36 research articles on this topic, 14 were non-empirical articles, 13 cross-sectional studies, 3 cohort studies, 2 pre-experimental designs (without control group), and a few other designs (case study, ecological study, “multiple studies” study, meta-analysis). Notable is the relatively high number of narrative reviews in this network, compared to the data-generating, empirical articles. Sixteen articles concluded that there was support for the association between swimming and asthma, ten articles concluded that there was no support for this association, and ten articles presented a mixed or an unclear conclusion (see Additional file 4).

**Table 1:** Characteristics of all 36 articles in chlorinated water network

		*N* publications	*n* citations (%) realized	*n* citations (%) not realized
Total		36	191 (34%)	379 (66%)
Article characteristics, study outcome	Category	*N* publications	*n* citations (%) realized	*n* citations (%) not realized
Authors’ conclusion	Positive	16	115 (42%)	161 (58%)
	Negative	10	47 (32%)	102 (68%)
	Mixed	5	17 (27%)	47 (73%)
Data-based conclusion	Positive	6	44 (48%)	48 (52%)
	Negative	16	116 (40%)	172 (60%)
	Mixed	0	0	0
Article characteristics, other content-related	Category	*N* publications	*n* citations (%) realized	*n* citations (%) not realized
Article type/study design	Narrative	14	31 (16%)	159 (84%)
	narrative review	11	18 (13%)	121 (87%)
	commentary	3	13 (26%)	38 (74%)
	Empirical	22	160 (42%)	220 (58%)
	cohort	3	10 (37%)	17 (63%)
	cross-sectional	13	91 (44%)	114 (56%)
	pre-experimental	2	16 (31%)	36 (69%)
	multiple designs	1	26 (76%)	8 (24%)
	ecological	1	9 (32%)	19 (68%)
	meta-analysis	1	8 (36%)	14 (64%)
	case study	1	0 (0%)	12 (100%)
Sample size (cat)	Low (1 – 199)	6	17 (20%)	69 (80%)
	Medium (200 – 1999)	8	68 (46%)	79 (54%)
	High ( ≥ 2000)	8	75 (51%)	72 (49%)
Study quality (cross-sectional)	Good	0	–	–
.	Fair	9	66 (46%)	77 (54%)
	Poor	4	25 (40%)	37 (60%)
Specificity	1 (non-specific)	5	0 (0%)	36 (100%)
	2	7	44 (31%)	96 (69%)
	3	9	46 (33%)	92 (67%)
	4	11	59 (38%)	96 (62%)
	5 (specific)	4	42 (42%)	59 (58%)
Article characteristics, not content-related	Category	*N* publications	*n* citations (%) realized	*n* citations (%) not realized
Language	English	33	191 (35%)	353 (65%)
	Other	3	0 (0%)	26 (100%)
Conclusive title	Not conclusive	25	124 (31%)	272 (69%)
	Conclusive	11	67 (39%)	107 (61%)
Funding source	Non-profit	15	111 (41%)	162 (59%)
	For-profit	1	8 (36%)	14 (64%)
	Both	5	42 (52%)	39 (48%)
	Not reported	15	30 (15%)	164 (85%)
Number of authors	1 – 2	10	24 (18%)	106 (82%)
	3 – 4	11	51 (31%)	114 (71%)
	5 – 6	7	40 (49%)	41 (51%)
	≥ 7	8	76 (39%)	118 (61%)
Number of affiliations	1	15	54 (25%)	161 (75%)
	2	10	48 (36%)	87 (74%)
	≥ 3	11	89 (40%)	131 (60%)
Number of references	< 25	10	18 (14%)	113 (86%)
	25 – 40	15	108 (40%)	165 (60%)
	≥ 40	11	65 (39%)	101 (61%)
Journal impact factor (cat)	0 – 2	10	59 (34%)	116 (66%)
	2 – 4	13	33 (22%)	117 (78%)
	≥ 4	12	99 (44%)	125 (56%)
Author characteristics	Category	*N* publications	*n* citations (%) realized	*n* citations (%) not realized
Gender	Male	24	148 (35%)	269 (65%)
	Female	12	43 (28%)	110 (72%)
Country	Belgium	12	112 (44%)	143 (56%)
	Other North West Europe	8	22 (24%)	71 (76%)
	UK	3	7 (14%)	44 (86%)
	Germany	2	11 (44%)	14 (56%)
	Netherlands	1	4 (57%)	3 (43%)
	Norway	1	0 (0%)	10 (100%)
	Sweden	1	0	0
	South Europe	12	32 (23%)	105 (77%)
	Italy	5	15 (19%)	65 (81%)
	Spain	4	17 (43%)	23 (58%)
	France	2	0 (0%)	5 (100%)
	Croatia	1	0 (0%)	12 (100%)
	North America	4	25 (29%)	60 (71%)
	USA	3	17 (30%)	39 (70%)
	Canada	1	8 (28%)	21 (72%)
Type of affiliation	University	27	154 (34%)	303 (66%)
	Government	2	8 (25%)	24 (75%)
	Industry	1	1 (8%)	11 (92%)
	Other	6	28 (41%)	41 (59%)
Citation characteristics	category		*n* citations (%) realized	*n* citations (%) not realized
Time to citation (in years)	0 – <1		26 (29%)	63 (71%)
	1 – <2		42 (40%)	64 (60%)
	2 – <3		30 (37%)	52 (63%)
	3 – <4		23 (29%)	55 (71%)
	4 – <5		24 (39%)	38 (61%)
	5 – <6		16 (35%)	30 (65%)
	6 – <7		12 (29%)	30 (71%)
	7 – <8		7 (26%)	20 (74%)
	≥ 8		11 (29%)	27 (71%)
Authority	0 – 5		41 (23%)	134 (77%)
	6 – 50		105 (37%)	177 (63%)
	≥ 51		45 (40%)	68 (60%)
Self-citation	No		137 (28%)	349 (72%)
	Yes		54 (64%)	30 (36%)

A ranking of the most cited articles and authors can be found in Table 2. Articles were cited between 0 and 26 times (with a median of 4). It seems that Bernard’s article from 2003 is generally seen as the first study that started this line of research [10]. There was an earlier publication in 2002 [16], but asthma was not explicitly mentioned, and one of the studies reported in this publication was also reported in the 2003 publication. (Both publications reported on multiple studies.) A more recent publication, by Font-Ribera, was cited 11 times out 12 potential citations; in other words, almost all subsequent articles cited this publication [35]. The table further shows that, within this line of research, Bernard is clearly the most cited author. It also shows that 5 out of 6 researchers with the highest authority are working at the same research group.

**Table 2:** Top 6 of articles (above) and authors (below) within network, based on the number of received citations up to 2016

Article rank	Article’s first author	Title	Year	No. of received citations (% of potential citations)
1	Bernard	Lung hyperpermeability and asthma	2003	26 (76%)
2	Bernard	Chlorinated pool attendance	2006	20 (71%)
3	Bernard	Infant swimming practice	2007	18 (69%)
4	Schoefer	Health risks of early swimming	2008	13 (59%)
5	Carbonelle	Changes in serum pneumoproteins	2002	12 (35%)
6	Font-Ribera	Swimming pool attendance	2011	11 (92%)
Author rank	Author	Affiliation	Country	No. of received citations (= authority)
1	A. Bernard	Catholic University of Louvain, Brussels	Belgium	112
2	S. Carbonelle	Catholic University of Louvain, Brussels	Belgium	79
3	C. de Burbure	Catholic University of Louvain, Brussels	Belgium	62
4	M. Nickmilder	Catholic University of Louvain, Brussels	Belgium	59
5	O. Michel	Free University of Brussels, Brussels	Belgium	57
6	X. Dumont	Catholic University of Louvain, Brussels	Belgium	55

The results from the regression analyses of citation are shown in Table 3. We used odds ratios for the interpretation of the results. An odds ratio greater than 1 signifies an increased likelihood of citation. Originally, we had planned to adjust for article type and sample size as we consider both of them to be justified determinants of citation. However, article type and sample size were highly related and adjusting for both led to unstable results. Therefore, we only adjusted for article type.

**Table 3:** Odds ratios (95% CIs) for the chance of being cited, all types of articles included, *N* = 36, *n* = 570)

Article characteristics, study outcome	Crude OR	Adjusted OR^a^
Authors’ conclusion (pos vs neg)	1.4 (0.9–2.3)	1.8 (1.1–2.9)
Data-based conclusion (pos vs neg)^b^	1.4 (0.8–2.3)	–
Article characteristics, other content-related	Crude OR	Adjusted OR^a^
Article type (empirical vs narrative)	4.2 (2.6–6.7)	–
Sample size (ref: low)^b^		
medium	4.2 (2.1–8.4)	–
high	5.8 (2.9–11.6)	–
Study quality (fair vs poor)^c^	1.6 (0.7–3.2)	1.4 (0.6–3.0)
Specificity (cont)	1.3 (1.1–1.6)	1.4 (1.2–1.7)
Article characteristics, not content-related	Crude OR	Adjusted OR^a^
Language	^d^	^d^
Conclusive title (yes vs no)	1.3 (0.9–2.0)	1.2 (0.8–1.8)
Funding source (ref: exclusively non-profit)		
profit or both profit/non-profit	1.6 (0.9–2.6)	1.6 (0.9–2.7)
not reported	0.2 (0.1–0.4)	0.3 (0.2–0.5)
Number of authors (ref: 1 – 2)		
3 – 4	2.2 (1.2–4.0)	2.1 (1.2–4.0)
5 – 6	6.0 (3.0–11.9)	3.2 (1.5–6.6)
≥ 7	2.9 (1.6–5.1)	1.7 (0.9–3.2)
Number of affiliations (ref: 1)		
2	2.0 (1.2–3.3)	1.7 (1.0–2.9)
≥ 3	2.2 (1.4–3.4)	1.4 (0.9–2.3)
Number of references (ref: < 25)		
25 – 40	4.7 (2.6–8.6)	3.3 (1.7–6.1)
≥ 40	5.1 (2.7–9.6)	5.1 (2.6–9.8)
Journal impact factor (ref: 0 – 2)		
2 – 4	0.5 (0.3–0.9)	0.7 (0.4–1.1)
≥ 4	1.9 (1.2–3.1)	2.0 (1.2–3.3)
Author characteristics	Crude OR	Adjusted OR^a^
Gender (female vs male)	0.8 (0.5–1.2)	0.6 (0.4–0.9)
Country (ref: Belgium)		
Other North West Europe	0.8 (0.4–1.7)	0.9 (0.4–1.9)
South Europe	0.4 (0.2–0.7)	0.4 (0.2–0.7)
North America	0.4 (0.2–1.0)	0.3 (0.1–0.7)
Type of affiliation (other vs university)	1.1 (0.7–1.8)	0.8 (0.5–1.4)
Citation characteristics	Crude OR	Adjusted OR^a^
Time to citation (cont, in years)	1.0 (1.0–1.1)	1.0 (0.9–1.1)
Authority (ref: low)		
medium	2.4 (1.5–4.0)	2.8 (1.7–4.6)
high	4.1 (2.2–7.8)	4.1 (2.1–8.0)
Self-citation (yes vs no)^e^	4.6 (2.8–7.5)	5.2 (3.1–8.8)

*N* number of articles, *n* number of potential citation paths

^a^Adjusted for article type (categories: non-empirical vs empirical)

^b^Only for empirical studies

^c^Only for cross-sectional studies

^d^Model did not converge

^e^Analyzed with fixed model logistic regression

First of all, if we look at the *content-related article characteristics*, there was some evidence for citation bias within this network: articles with a positive authors’ conclusion were cited more often than articles with a negative conclusion (odds ratio (OR) 1.8, 95% confidence interval (CI) 1.1–2.9). The data-based conclusion did not seem to impact the chance of citation. Articles with an empirical research design were cited more often than non-empirical ones (OR 4.2, CI 2.6–6.7), and a higher sample size was associated with a higher number of citations (OR 5.8, CI 2.9–11.6). Articles that specifically focused on our hypothesis under investigation, and thus fitted well in our network, were cited more often than those that did not (one step increase in specificity: OR 1.4, CI 1.2–1.7). However, the quality of the cross-sectional studies did not seem to have an impact on citation.

Other *article characteristics* that increased the number of citations were journal impact factor, number of authors, number of references, and reporting of a funding statement. *Author characteristics* that showed an impact on the number of citations were gender and region. The *within-network authority* of the cited article also increased the likelihood of citation (OR 4.1, CI 2.1–8.0), and there was strong evidence for *self-citation* in this network (OR 5.2, CI 3.1–8.8).

In addition, we tested whether articles with similar characteristics were more likely to cite each other. The results showed that concordance between articles did not have much impact on the likelihood of being cited, except possibly for concordance of study quality (Additional file 5).

We performed post hoc sensitivity analyses in which we excluded the narrative reviews and commentaries (with sample size being equal to 0) and adjusted for both study design and sample size (Additional file 6). The results were very similar to those of the complete data set. We performed an additional analysis in which we also excluded the ecological study and the case study (both with a wildly varying sample size). These results did show some stronger evidence for citation bias as studies with positive authors’ conclusions and data-based conclusions accumulate about three times more citations (Additional file 6). The results on the other determinants were fairly consistent with the previous findings.

The occurrence of citation bias was tested with two operationalizations of study outcome. The authors’ conclusion on the relation between swimming and asthma, as was often stated in the abstract, seemed to have an impact on citation, while the conclusion based on the underlying data seemed to have no impact. This implied that the authors’ conclusion and the data-based conclusion did not always concur. A post hoc chi square test showed that 40% of the negative results were interpreted as positive by the authors of those articles, while 0% of the positive results were interpreted as negative (data not shown; *χ* ^*2*^ (1) = 6.3, *p* = 0.012).

**Fig. 1:**
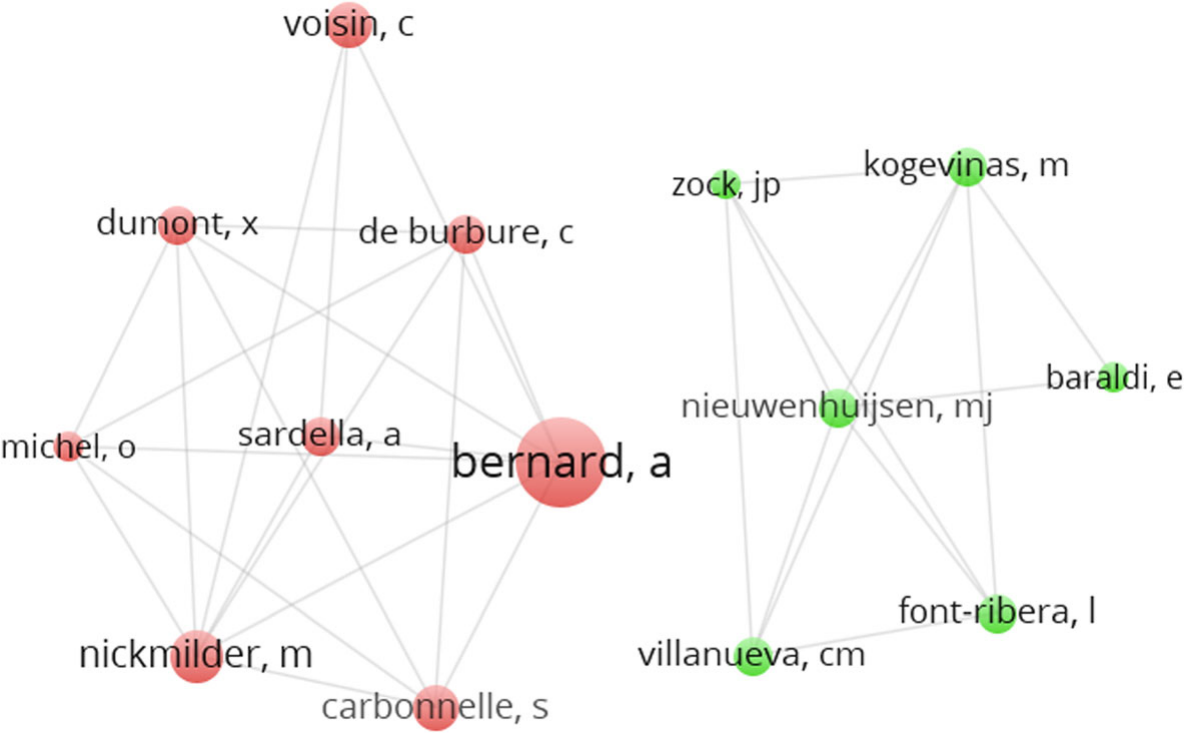
Network visualization - authors. Each *circle* represents an author. The bigger the circle, the higher the number of publications. Each *line* represents a shared authorship. This graph shows that there are two main research groups active in this network. Authors with less than three publications were excluded

Also, because self-citation seemed abundant in this field, we wondered whether particular researchers or research groups were responsible for this. First we assessed the number of research groups based on co-author relations of authors with at least three publications. Two research groups could be distinguished (Fig. 1). We then coded group membership for each article depending on which groups the authors belong to. Twelve articles stemmed from Bernard’s research group, nine articles came from the other identified research group, and 15 articles were written by authors that belonged to neither group. There were no articles in which the authors from both research groups collaborated. Interestingly, 11 of the 12 articles from Bernard’s research group had a positive authors’ conclusion, whereas the other research group only published articles with a negative or mixed/unclear conclusion (Additional file 7). Stratified logistic regression analyses showed that, indeed, self-citation occurred in both research groups (Additional file 7). This finding was confirmed by the self-citation rates of individual authors: the top 6 of self-citing authors stem from both of the research groups (Additional file 7).

## Discussion

Our research aim was to find out which determinants have an impact on the likelihood of being cited in the literature on swimming in chlorinated water and childhood asthma. We found that self-citation, large sample size, and empirical articles play a major role: in this network, they lead to a four to six times higher odds for citation. Other determinants associated with double citation odds or higher are journal impact factor, reporting of funding information, number of authors, authority, geographical region, and number of references.

There is some evidence for citation bias in this field of research. Articles in which the authors concluded that swimming in chlorinated water and asthma were related, are cited more often than those that did not. However, this finding is not robust and depends on the statistical test performed. Similarly, when we looked at the data-based conclusion rather than the authors’ conclusion, it did not have much impact on citation. This suggests that citation bias, if it occurs, is mostly based on interpretations and not so much on the underlying data. This would be in line with previous findings from our meta-analysis on citation bias, which showed that the authors’ conclusion has a higher impact on the likelihood of being cited than a data-based conclusion [2]. Still, the impact of study outcome on citation in the current network is small and not robust.

One explanation why we did not find strong evidence for citation bias might be due to the small size of the network. It is easy to have an overview of all published work in this field, without relying on the people that one knows, or on references that one happens to find. Also, this network seems to be quite balanced between proponents and opponents of the pool chlorine hypothesis, if we look at the number of positive [15] and negative articles [10]. This is contrary for instance, to the field of trans fatty acids and cholesterol, where 83% of all the published literature supports the view of an association, and citation bias was found to be much more prevalent in this field (Urlings et al.: Citation bias in the trans fatty acid literature: evidence from a citation network analysis on the effect of industrially produced trans fat and cholesterol, submitted). Such balance in viewpoints secures the possibility of adequate contradiction so that no point of view disappears until the debate is settled.

This debate, on whether swimming in chlorinated water increases the chance of developing asthma later during childhood, could best be settled by means of longitudinal research. Nevertheless, the majority of empirical articles used a cross-sectional design, and one third of all articles on this topic is not even empirical. We identified only three prospective cohort studies within the network [35, 36, 46], one of which did not assess asthma per se but lung function in general. Of the remaining two studies, Voison et al. concluded that they had found support for the positive association between swimming and asthma development [46], whereas Font-Ribera et al. concluded the opposite that there was a smaller chance of current asthma for children who had swum more [35].

Our citation analysis has several limitations. First of all, the network is small relative to the number of predictors, which makes it vulnerable for chance findings. Similarly, some research design categories were represented by only one publication. Secondly, there was a high degree of multicollinearity between study design and sample size, which made it impossible to adjust for all the factors we had originally planned. We dealt with these two limitations by running several sensitivity analyses, with different adjustments and with inclusion of different research designs. Thirdly, asthma is difficult to diagnose, and in the literature, we found different ways to assess asthma, with a varying degree of validity. To make these articles compatible, we combined these different assessments into one measure for data-based conclusion on asthma.

Our network consists of publications identified by the Web of Science Core Collection. This database offers the option to download reference lists as part of the search output. Other databases, such as MEDLINE and Embase, do not provide this option yet. Because these reference lists are needed by the software we used (CitNetExplorer) to build a citation network, we did not include these other databases in our search strategy. It is therefore likely that we missed parts of the literature. However, we see no reason why the citation dynamics in the Web of Science Core Collection would be different from other databases. Nevertheless, it would be an improvement if reference lists were added to the search output of these other databases so that this literature can be included in future citation networks.

## Conclusions

There is clear evidence of selective citation in the research field studying the relation between swimming in chlorinated water and childhood asthma: several factors have an impact on the chance of being cited. Authors particularly prefer to cite their own work over those of other authors on the same topic. The evidence for citation bias in this field was not very strong and inconsistent. The impact of citation bias on the development of knowledge therefore seems limited in this field.

## Additional file

Additional file 1: Protocol deviations. (DOCX 126 kb)
Additional file 2: Search strategy. (DOCX 159 kb)
Additional file 3: Flow diagram of search output. (DOCX 143 kb)
Additional file 4: Network visualization - publications. (DOCX 388 kb)
Additional file 5: Concordance analyses. (DOCX 121 kb)
Additional file 6: Sensitivity analyses.. (DOCX 67.8 kb)
Additional file 7: Research groups and self-citation. (DOCX 95.9 kb)

## Abbreviations

CI: 95% confidence interval
DOI: Digital object identifier
JCR: Journal Citation Reports
*N*: Number of articles
*n*: Number of potential citation paths
NCl_3_: Trichloramine/nitrogen trichloride
NIH: National Institutes of Health (in the USA)
OR: Odds ratio
