# Supplementary material for: Selective citation in the literature on swimming in chlorinated water and childhood asthma: a network analysis
Source: Res Integr Peer Rev. 2017 Oct 2;2:17. doi: 10.1186/s41073-017-0041-z (PMC5803637; doi:10.1186/s41073-017-0041-z)
Supplement: Additional file 1: — Protocol deviations. (DOCX 126 kb) [file 41073_2017_41_MOESM1_ESM.docx]

**Selective citation in the literature on swimming in chlorinated water and childhood asthma: a network analysis**

**Additional file 1: Protocol deviations**

**inclusion and exclusion criteria.** We made some small changes to the inclusion and exclusion criteria. Here is the updated table.

| Inclusion criteria |
| --- |
| Publications assessing the relation between swimming in indoor chlorinated pools and asthma. |
| Study design is observational (cross-sectional, cohort, case control, ecological, case study), experimental, review or other type of synthesis paper. |
| All languages. |
| Asthma can be assessed in several ways in these publications: by doctor’s diagnosis, self-assessment (or parents’ assessments), asthma-related symptoms, lung tests, and blood biomarkers. |
| Exclusion criteria |
| Swimming pool accidents. |
| Swimming as treatment for asthmatics. |
| Meeting abstracts, news items, no fulltext available. |
| Publications not including indoor chlorinated swimming pools. |
| Publications not including 18- swimmers, or in which 18- swimmers can not be clearly distuinguished from 18+ swimmers. |
| Publications based on non-swimmers, e.g. swimming pool workers. |

**specificity.** In the protocol it was stated to count the number of results in the abstract that were not related to the main claim of this network (i.e. the possible impact of swimming in chlorinated water on the development of asthma in children). During the scoring we realised this was not a good measure: it was difficult to score and did not express well what we wanted it to express: to what degree this article is about our hypothesis of interest. Instead we decided to subjectively score the article’s specificity based on based on the title. We did this in duplo to increase reliability.

**study outcome**. We changed the operationalization of study outcome. *Authors’ conclusion* stayed the same, but we combined the four data-based operationalizations of study outcome (asthma diagnosis, asthma symptoms, lung tests, blood tests) to one measure, called *data-based conclusion*. For each of the separate operationalizations a different set of articles was included in the analyses, making it difficult to compare and interpret the results. That is the reason why we combined those measures to one. In addition, we coded the type of *asthma assessment* as follows:

Asthma diagnosis by physician (1a. Current asthma; 1b. Combined measure of patients diagnosed with current asthma and patients scoring positive on another test such as EIB; 1c. Ever asthma)

Self-reported asthma diagnosis (2a. Current asthma; 2b. Combined measure of patients diagnosed with current asthma and patients scoring positive on another test such as EIB; 2c. Ever asthma)

Self-reported asthma symptoms (3a. Wheezing, short breath, chest tightness, lower respiratory symptoms; 3b. Coughing)

Lung tests (4.a Spirometry (FEV1, FVC, PEF, FEV1/FVC, etc); 4b. Trigger tests (EIB; metacholine challenge test); 4c. Inflammation (eNO);

Lung permeability tests (or blood tests; surfactant associated proteins such as SP-A, SP-B, SP-D, and clara cell CC16).

NB. ‘1’ is considered to be the best quality asthma assessment, ‘5’ the worst quality.

NB. If a study was on lung tests, but also presented asthma symptoms as demographic characteristics, we used the lung tests even though the asthma symptoms are higher in the hierarchy.

NB. If both raw and adjusted odds ratios are presented, we took the adjusted ones.

The *data-based conclusion* was coded as follows: 1. Significant positive association between swimming in chlorinated water and asthma; 2. No significant positive association (so no significant association, or a significant negative association); 3 Mixed (both positive and negative associations exists within the same type of *asthma assessment* category; 4. Unclear; 5. Not measured (e.g. in the case of narrative reviews).

Still, not all measurements are of the same quality (e.g. pneumoproteins in the blood are considered to be a biomarker for lung permeability, but it is not a validated measure for asthma). Therefore we used the hierarchy of *asthma assessment* for the scoring of the *data-based conclusion*.

E.g. If '1. Asthma diagnosis by physician’ showed a positive association with swimming, and the other health outcomes did not, then we scored the study outcome as ‘1. Significant positive association’. And if ‘1a. Current asthma’ showed no association, and ‘1b. Ever asthma’ did show a positive association, then we scored the study outcome as ‘2. No significant positive association’.

**journal impact factor**. We do not researchgate as our source for the journal impact factor. This does not seem to be valid, it deviates from other sources and it is unclear how it is calculated. Also, they sometimes have different impact factors for the same journal. Instead of researchgate, we decided to use the more offical JCR source.

**study quality**. We had not specified the quality checklist for cross-sectional studies. We decided to use the NIH NHLBI. This assessment does not yield a sum score, but a categorisation into good, fair, or poor.

**region.** Of the corresponding author, not of all authors.

**type of article / study design.** We classified the one systematic review on cross-sectional studies as an observational study. Also the one article with multiple designs, as most designs in this article were observational. Additionally we included a case study, and categorised it as an observational study. In our (adjusted) analyses, we discriminate between article type (empirical versus non-empirical articles) and study design. The study design refers to a more specific category within the article type. For example, the empirical article type can have several study designs, such as intervention study or observational study.

**time to citation.** In the protocol this was called publication time. It was stated that only citation paths with at least 1 year of publication time (i.e. the difference between the submission date of the potentially citing article and the publication date of the potentially cited article) would be included in the analyses. This 1 year embargo has been skipped, now all potential citation paths are included. We decided to do this because of a) higher power, b) already many realised citations within the first year (see Table 1).

**regression analyses.** Contrary to specified, we did not adjust for research quality, because: a) it did not show an impact on citation; b) it was only scored for cross-sectional studies (not enough studies in cohort / experimental design to include quality scores in the analyses); c) after the scoring experience, we had doubts about the validity of of this measure; d) it did not yield a sum score and the variation between studies is low. Sensititivy analyses showed that if we adjust for for research quality, the odds ratios are very similar.

In addition, and contrary to specified, we did not adjust for sample size in our main analyses (Table 3). Because non-empirical articles did not have a sample size, there was too much overlap between these variables to adjust for both simultaneously. However, we ran some sensitivity tests on the empirical articles in which we adjusted for both variables. In these sensitivity analyses we excluded the narrative reviews and commentaries as (potentially) cited articles from the regression analyses. The narrative reviews are a) an odd kind of article; b) have no sample size, leading to odd behaviour in the analyses when adjusting for sample size and research design.We also excluded the case study and the ecological study; of either type we only had 1 article, one with a sample size of 1 and the other with a sample size of almost 200,000. This would result in odd behaviour when it comes to the sample size adjusted analyses.

We did not exclude analyses based on less than 10 citation pathways.

**citing bias.** We did not calculate citing bias for this network.

**concordance.** We did not conduct concordance analyses for the complete list of determinants.
