# Supplementary material for: Selective citation in the literature on swimming in chlorinated water and childhood asthma: a network analysis
Source: Res Integr Peer Rev. 2017 Oct 2;2:17. doi: 10.1186/s41073-017-0041-z (PMC5803637; doi:10.1186/s41073-017-0041-z)
Supplement: Additional file 2: — Search strategy. (DOCX 159 kb) [file 41073_2017_41_MOESM2_ESM.docx]

**Selective citation in the literature on swimming in chlorinated water and childhood asthma: a network analysis**

**Additional file 2: Search strategy**

| determinant | health outcome | population |
| --- | --- | --- |
| "chlorinated" | "asthma*" | "child*" |
| "chlorine" | "atopy" | "adolescent*" |
| "chlorination" | "respiratory" | "boy*" |
| "swimming water" | "lung*" | "girl*" |
| "swimming pool*" | "bronch*" | "kid*" |
| "indoor pool*" | "pulmonary" | "toddler*" |
| "indoor swimming" | "wheez*" | "babies" |
| "NCl3" | "airway*" | "baby" |
| "nitrogen trichloride*" | "breath*" | "infant*" |
| "trichloramine*" | "cough*" | "young swimmer*" |
| "trihalomethane*" | "chest tightness" | "early life" |
| "trihaloacetic acid*" | "tight chest" | "early age" |
| "chloramine*" | "surfactant*protein*" |  |
|  | "SP-A" |  |
|  | "SP-B" |  |
|  | "IgE" |  |
|  | "pneumoprotein*" |  |
|  | "cc16" |  |
|  | "cc-16" |  |
|  | "clara cell*" |  |
|  | "eNO" |  |
|  | "exhaled NO" |  |
|  | "exhaled nitric oxide" |  |
|  | "EIB" |  |
|  | "EBC" |  |
|  | "exhaled breath condensate" |  |
|  | "methacholine challenge" |  |
|  | "eucapnic voluntary hyperventilation" |  |
|  | "EVH" |  |
|  | "FVC" |  |
|  | "vital capacity" |  |
|  | "FEV*" |  |
|  | "forced expiratory volume" |  |
|  | "Tiffeneau" |  |
