# Supplementary material for: Selective citation in the literature on swimming in chlorinated water and childhood asthma: a network analysis
Source: Res Integr Peer Rev. 2017 Oct 2;2:17. doi: 10.1186/s41073-017-0041-z (PMC5803637; doi:10.1186/s41073-017-0041-z)
Supplement: Additional file 4: — Network visualization - publications. (DOCX 388 kb) [file 41073_2017_41_MOESM4_ESM.docx]

**Selective citation in the literature on swimming in chlorinated water and childhood asthma: a network analysis**

**Additional file 4: Network visualization - publications**


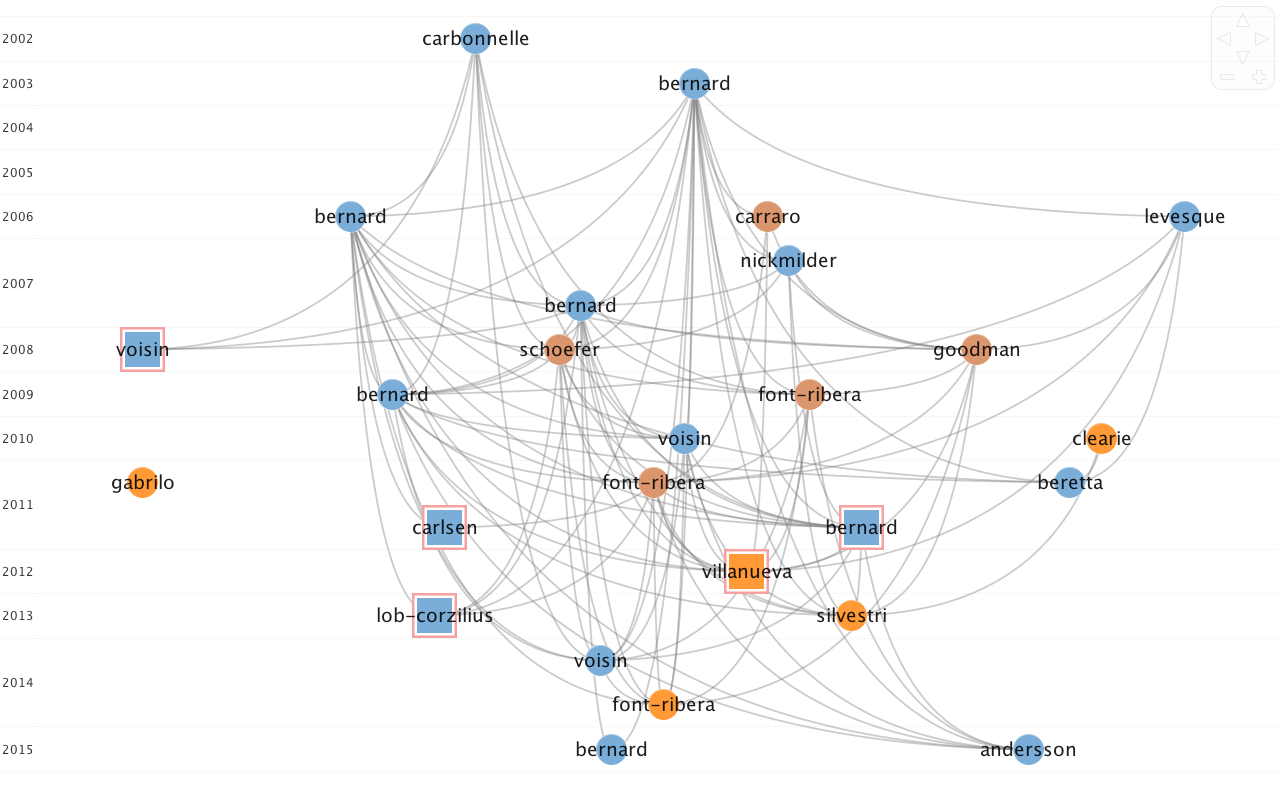


Blue circles represent articles with a positive authors’ conclusion. Orange circles represent articles with a negative authors’ conclusion. Articles with a neutral conclusion are not depicted in this graph. The lines between articles represent (realized) citations (with the newest article citing the oldest article). The y-axis represents the publication year, with top being the oldest and bottom the newest. The x-axis does not bear any meaning. The circles represent empirical articles, the squares represent narrative reviews and commentaries.
