# Supplementary material for: Selective citation in the literature on swimming in chlorinated water and childhood asthma: a network analysis
Source: Res Integr Peer Rev. 2017 Oct 2;2:17. doi: 10.1186/s41073-017-0041-z (PMC5803637; doi:10.1186/s41073-017-0041-z)
Supplement: Additional file 5: — Concordance analyses. (DOCX 121 kb) [file 41073_2017_41_MOESM5_ESM.docx]

**Selective citation in the literature on swimming in chlorinated water and childhood asthma: a network analysis**

**Additional file 5: Concordance analyses**

**Table A5.1. Concordance odds ratios (95% CI’s) for the chance of being cited, all types of articles included, N = 36, n = 570)**

| **Content-related characterstics** | **Crude OR** | **Adjusted OR *** |
| --- | --- | --- |
| Authors’ Conclusion (conc. vs. not) | 1.5 (0.9 – 2.4) | 1.6 (1.0 – 2.6) |
| Data-based Conclusion (conc. vs. not) | 1.2 (0.7 – 2.0) | 1.2 (0.7 – 2.0) |
| Article Type (conc. vs. not) | 1.4 (1.0 – 1.9) | - |
| Study Quality (conc. vs. not) | 3.0 (1.1 – 8.1) | - |
|  |  |  |
| **Not content-related characteristics** | **Crude OR** | **Adjusted OR *** |
| Conclusive Title (conc. vs. not) | 1.1 (0.8 – 1.6) | 1.2 (0.8 – 1.7) |
| Type of Affiliation (conc. vs. not) | 1.8 (0.9 – 3.7) | 2.1 (1.0 – 4.4) |
| Gender (conc. vs. not) | 1.1 (0.8 – 1.6) | 1.1 (0.8 – 1.6) |

* adjusted for article type (categories: non-empirical (narrative reviews and commentaries) vs empirical (cross-sectional, experimental, cohort, case, ecological, multiple, meta-analysis). N: number of articles. n: number of potential citation paths

**Table A5.2. Concordance odds ratios (95% CI’s) for the chance of being cited, without narrative reviews and commentaries (as cited articles, N = 22, n = 380)**

| **Content-related characterstics** | **Crude OR** | **Adjusted OR *** | | |
| --- | --- | --- | --- | --- |
| Authors’ Conclusion (conc. vs. not) | 1.7 (1.0 – 2.8) | 1.7 (1.0 – 2.8) |  |  |
| Data-based Conclusion (conc. vs. not) | 1.2 (0.7 – 2.0) | 1.3 (0.8 – 2.4) |  |  |
| Study Design (conc. vs. not) *** | 2.8 (1.3 – 6.4) | 2.3 (0.9 – 5.4) ** |  |  |
| Study Quality (conc. vs. not) | 3.0 (1.1 – 8.1) | 2.9 (1.1 – 7.9) ** |  |  |
|  |  |  | |  |
| **Not content-related characteristics** | **Crude OR** | **Adjusted OR *** | |  |
| Conclusive Title (conc. vs. not) | 1.2 (0.8 – 1.8) | 1.2 (0.8 – 1.8) |  |  |
| Type of Affiliation (conc. vs. not) | 1.7 (0.8 – 3.8) | 1.8 (0.8 – 4.0) |  |  |
| Gender (conc. vs. not) | 1.2 (0.8 – 1.7) | 1.2 (0.8 – 1.8) |  |  |

* adjusted for study design (obs vs exp) and log sample size. ** adjusted for log sample size. *** without narrative citing articles as well (N = 20, n = 213), conc: both observational or both experimental. N: number of articles. n: number of potential citation paths
