# Supplementary material for: Selective citation in the literature on swimming in chlorinated water and childhood asthma: a network analysis
Source: Res Integr Peer Rev. 2017 Oct 2;2:17. doi: 10.1186/s41073-017-0041-z (PMC5803637; doi:10.1186/s41073-017-0041-z)
Supplement: Additional file 6: — Sensitivity analyses.. (DOCX 67.8 kb) [file 41073_2017_41_MOESM6_ESM.docx]

**Selective citation in the literature on swimming in chlorinated water and childhood asthma: a network analysis**

**Additional file 6: Sensitivity analyses**

**Table A6.1. Odds ratios (95% CI’s) for the chance of being cited, without narrative reviews and commentaries (N = 22, n = 380)**

| **Article characteristics,**  **study outcome** | **Crude OR** | **Adjusted OR *** |
| --- | --- | --- |
| Authors’ Conclusion (pos vs neg) | 1.8 (1.1 – 2.8) | 1.8 (1.1 – 2.9) |
| Data-based Conclusion (pos vs neg) | 1.4 (0.8 – 2.3) | 1.0 (0.5 – 1.7) |
|  |  |  |
| **Article characteristics,**  **other content-related** | **Crude OR** | **Adjusted OR *** |
| Study Design (exp vs observational) | 0.5 (0.2 – 0.9) | - |
| Sample Size (ref: low) |  |  |
| medium | 4.2 (2.1 – 8.4) | 34.5 (4.4 – 274) *** |
| high | 5.8 (2.9–11.6) | 47.3 (6.0 – 375) *** |
| Study Quality (fair vs poor) **** | 1.6 (0.7 – 3.2) | 1.4 (0.6 – 3.0) ** |
| Specificity (cont) | 1.4 (1.2 – 1.7) | 1.3 (1.0 – 1.6) |
|  |  |  |
| **Article characteristics,**  **not content-related** | **Crude OR** | **Adjusted OR *** |
| Language | - | - |
| Conclusive Title (yes vs no) | 1.0 (0.6 – 1.7) | 1.0 (0.6 – 1.8) |
| Funding Source (ref: exclusively non-profit) |  |  |
| profit or both profit/non-profit | 1.3 (0.7 – 2.3) | 1.2 (0.6 – 2.2) |
| not reported | 0.2 (0.1 – 0.4) | 0.1 (0.1 – 0.3) |
| Number of Authors (ref: 1-2) |  |  |
| 3 - 4 | 2.1 (0.9 – 4.8) | 13.9 (4.1 – 47.3) |
| 5 - 6 | 2.6 (1.2 – 6.0) | 13.5 (4.3 – 41.9) |
| >= 7 | 1.3 (0.6 – 2.6) | 6.8 (2.4 – 19.6) |
| Number of Affiliations (ref: 1) |  |  |
| 2 | 1.5 (0.8 – 2.8) | 1.6 (0.8 – 3.2) |
| >= 3 | 1.2 (0.7 – 2.1) | 1.3 (0.7 – 2.3) |
| Number of References (ref: <25) |  |  |
| 25 - 40 | 4.0 (1.9 – 8.6) | 3.1 (1.4 – 7.0) |
| >= 40 | 7.9 (3.4–18.8) | 6.2 (2.5 – 15.2) |
| Journal Impact Factor (ref: 0-2) |  |  |
| 2 – 4 | 0.7 (0.4 – 1.4) | 0.4 (0.2 – 0.8) |
| >= 4 | 1.6 (0.9 – 2.7) | 1.5 (0.9 – 2.6) |
|  |  |  |
| **Author characteristics** | **Crude OR** | **Adjusted OR *** |
| Gender (female vs male) | 0.7 (0.4 – 1.2) | 0.8 (0.5 – 1.3) |
| Country (ref: Belgium) |  |  |
| Other North West Europe | 1.1 (0.5 – 2.7) | 0.9 (0.4 – 2.2) |
| South Europe | 0.3 (0.2 – 0.5) | 0.2 (0.1 – 0.5) |
| North America | 0.2 (0.1 – 0.7) | 0.2 (0.1 – 0.5) |
| Type of Affiliation (other vs university) | 0.9 (0.5 – 1.6) | 0.7 (0.4 – 1.3) |
|  |  |  |
| **Citation characteristics** | **Crude OR** | **Adjusted OR *** |
| Time to Citation (cont, in years) | 1.0 (0.9 – 1.1) | 1.0 (0.9 – 1.2) |
| Authority (ref: low) |  |  |
| medium | 3.3 (1.9 – 5.9) | 2.9 (1.6 – 5.4) |
| high | 4.7 (2.2–10.1) | 4.4 (1.9 – 9.8) |
| Self-citation (yes vs no) ***** | 5.4 (2.8 – 10.3) | 5.5 (2.8 – 10.6) |

* adjusted for study design (experimental versus observational) and log sample size. ** adjusted for log sample size *** adjusted for study design. ******** only for cross-sectional studies. ***** analyzed with fixed model logistic regression. N: number of articles. n: number of potential citation paths

**Table A6.2. Odds ratios (95% CI’s) for the chance of being cited, without narrative reviews, commentaries, ecological study, and case study (because of uncomparable sample size adjustment, N = 20, n = 340)**

| **Article characteristics,**  **study outcome** | **Crude OR** | **Adjusted OR *** |
| --- | --- | --- |
| Authors’ Conclusion (pos vs neg) | 2.4 (1.4 – 3.9) | 3.5 (1.9 – 6.2) |
| Data-based Conclusion (pos vs neg) | 3.7 (1.8 – 7.5) | 2.4 (1.1 – 5.2) |
|  |  |  |
| **Article characteristics,**  **other content-related** | **Crude OR** | **Adjusted OR *** |
| Study Design (exp vs observational) | 0.4 (0.2 – 0.8) | - |
| Study Design (ref: cross-sect) |  |  |
| cohort | 0.7 (0.3 – 1.7) | - |
| experimental | 0.4 (0.2 – 0.8) | - |
| Sample Size (ref: low) |  |  |
| medium | 3.4 (1.7 – 7.0) | 22.2 (2.7 – 183) *** |
| high | 6.1 (2.9–12.8) | 39.1 (4.7 – 326) *** |
| Study Quality (fair vs poor) **** | 1.6 (0.7 – 3.2) | 1.4 (0.6 - 3.0) ** |
| Specificity (cont) | 1.8 (1.5 – 2.3) | 1.7 (1.2 – 2.2) |
|  |  |  |
| **Article characteristics,**  **not content-related** | **Crude OR** | **Adjusted OR *** |
| Language | - | - |
| Conclusive Title (yes vs no) | 1.7 (1.0 – 2.9) | 2.3 (1.2 – 4.4) |
| Funding Source (ref: exclusively non-profit) |  |  |
| profit or both profit/non-profit | 1.3 (0.7 – 2.3) | 1.1 (0.6 – 2.1) |
| not reported | 0.2 (0.1 – 0.5) | 0.2 (0.1 – 0.6) |
| Number of Authors (ref: 1-2) |  |  |
| 3 - 4 | 1.6 (0.5 – 4.7) | 5.6 (1.6–19.3) |
| 5 - 6 | 3.0 (1.0 – 9.2 | 4.3 (1.4–13.6) |
| >= 7 | 1.0 (0.4 – 2.8) | 2.1 (0.7 - 6.4) |
| Number of Affiliations (ref: 1) |  |  |
| 2 | 0.9 (0.4 – 1.8) | 0.7 (0.3 – 1.6) |
| >= 3 | 0.7 (0.4 – 1.4) | 0.5 (0.2 – 0.9) |
| Number of References (ref: <25) |  |  |
| 25 - 40 | 3.2 (1.5 – 7.1) | 2.4 (1.1 – 5.5) |
| >= 40 | 5.8 (2.4–13.8) | 3.9 (1.5 – 9.8) |
| Journal Impact Factor (ref: 0-2) |  |  |
| 2 – 4 | 1.2 (0.5 – 2.6) | 0.9 (0.4 – 2.1) |
| >= 4 | 1.6 (0.9 – 2.7) | 1.7 (1.0 – 3.0) |
|  |  |  |
| **Author characteristics** | **Crude OR** | **Adjusted OR *** |
| Gender (female vs male) | 0.8 (0.5 – 1.3) | 0.5 (0.3 – 0.9) |
| Country (ref: Belgium) |  |  |
| Other North West Europe | 0.9 (0.4 – 2.2) | 0.2 (0.1 – 0.7) |
| South Europe | 0.3 (0.1 – 0.5) | 0.2 (0.1 – 0.4) |
| North America | 0.2 (0.1 – 0.6) | 0.1 (0.0 – 0.3) |
| Type of Affiliation (other vs university) | 0.8 (0.5 – 1.3) | 0.3 (0.2 – 0.6) |
|  |  |  |
| **Citation characteristics** | **Crude OR** | **Adjusted OR *** |
| Time to Citation (cont, in years) | 1.0 (0.9 – 1.1) | 1.1 (1.0 – 1.2) |
| Authority (ref: low) |  |  |
| medium | 3.2 (1.7 – 5.8) | 3.2 (1.6 – 6.3) |
| high | 5.2 (2.3 – 12.0 ) | 9.2 (3.3 – 25.8) |
| Self-citation (yes vs no) ***** | 7.5 (3.5 – 15.9) | 8.9 (4.0 – 19.7) |

* adjusted for study design (experimental versus observational) and log sample size. ** adjusted for log sample size *** adjusted for study design. ******** only for cross-sectional studies. ***** analyzed with fixed model logistic regression. N: number of articles. n: number of potential citation paths
