# Supplementary material for: Selective citation in the literature on swimming in chlorinated water and childhood asthma: a network analysis
Source: Res Integr Peer Rev. 2017 Oct 2;2:17. doi: 10.1186/s41073-017-0041-z (PMC5803637; doi:10.1186/s41073-017-0041-z)
Supplement: Additional file 7: — Research groups and self-citation. (DOCX 95.9 kb) [file 41073_2017_41_MOESM7_ESM.docx]

**Selective citation in the literature on swimming in chlorinated water and childhood asthma: a network analysis**

**Additional file 7: Research groups and self-citation**

**Table A7.1. Research group and authors’ conclusion.**

| **Authors’ conclusion** | **Research group** | | | **Total** |
| --- | --- | --- | --- | --- |
|  | **A** | **B** | **C** |  |
| **Positive** | 5 | 11 | 0 | 16 |
| **Negative** | 5 | 0 | 5 | 10 |
| **Mixed/Unclear** | 5 | 1 | 4 | 10 |
| **Total** | **15** | **12** | **9** | 36 |

**Note**. Research group A: did not stem from any of the identified research groups, B: stemmed from Bernard’s research group, C: stemmed from the other identified research group. *χ^2^* (4) = 19.1, *p* = 0.001).

**Table A7.2. Self-citation odds ratios for the chance of being cited, stratified by research group.**

| **Self-citation (yes vs. no)** | **Crude OR** | **Adjusted OR *** |
| --- | --- | --- |
| Group A | ** | ** |
| Group B | 8.4 (3.6 – 19.4) | 10.9 (3.8 – 31.5) |
| Group C | 3.3 (1.0 – 10.8) | ** |
| Unstratified | 4.6 (2.8 – 7.5) | 5.2 (3.1 – 8.8) |

**Note.** Research group A: did not stem from any of the identified research groups, B: stemmed from Bernard’s research group, C: stemmed from the other identified research group.* adjusted for study design (obs vs exp) and log sample size. ** did not converge. Analyzed with fixed-effects logistic regression. Stratification was based on the research group of the *citing* article.

**Table A7.3. Top 6 of authors within network, based on the number of self-citations up to 2016.**

| **Author rank** | **Author** | **Affiliation** | **Country** | **Self-citation rate ***  **(realized / potential self-citations)** |
| --- | --- | --- | --- | --- |
| 1 | A. Sardella | Catholic University of Louvain, Brussels | Belgium | 2.5 (5/5) |
| 2 | M. Kogevinas | Centre for Research in Environmental Epidemiology, Barcelona | Spain | 1.8 (5/6) |
| 3 | L. Font-Ribera | Centre for Research in Environmental Epidemiology, Barcelona | Spain | 1.8 (5/6) |
| 4 | C. Villanueva | Centre for Research in Environmental Epidemiology, Barcelona | Spain | 1.8 (5/6) |
| 5 | A. Bernard | Catholic University of Louvain, Brussels | Belgium | 1.7 (37/63) |
| 6 | M. Nickmilder | Catholic University of Louvain, Brussels | Belgium | 1.6 (7/14) |

**Note.** * The self-citation rate of an author states the odds of citing potential papers of oneself (realized self-citations / potential self-citations) relative to the total odds (total realized citations / total potential citations). Authors with less than 5 self-citations were excluded.
